# Placental extract suppresses cardiac hypertrophy and fibrosis in an angiotensin II-induced cachexia model in mice

**DOI:** 10.1016/j.heliyon.2019.e02655

**Published:** 2019-10-13

**Authors:** Akihiro Yamauchi, Akiko Kamiyoshi, Takayuki Sakurai, Hiroyuki Miyazaki, Eiichi Hirano, Hong Seok Lim, Taiichi Kaku, Takayuki Shindo

**Affiliations:** a Department of Cardiovascular Research, Shinshu University Graduate School of Medicine, Matsumoto, Japan; b Japan Bio Products Co., Ltd., Tokyo, Japan

**Keywords:** Biochemistry, Cardiac hypertrophy, Human placenta extract, Cardiac fibrosis, Angiotensin II, Cachexia

## Abstract

Cachexia is an intractable metabolic disorder that causes extreme weight loss. It is a symptom of many chronic diseases, including cancer, liver failure, congestive heart failure and chronic kidney disease, and there is as yet no effective treatment. While the mechanisms underlying cachexia are complex, it is often accompanied by elevated angiotensin II (Ang II). Human placental extract (HPE) is a source of numerous biologically active molecules and has been used clinically to treat chronic hepatitis, liver cirrhosis and other chronic diseases. Here, we investigated the effects of HPE in an Ang II-induced cachexia model in mice. HPE treatment preserved both fat mass and lean body mass and suppressed weight loss in the cachexia model, though food intake was unaffected. Ang II infusion also caused cardiac hypertrophy and fibrosis. HPE suppressed these effects as well as Ang II-induced cardiac expression of genes related to heart failure and cardiac remodeling. HPE also reversed Ang II-induced downregulation of mitochondria-related molecules and suppressed cardiac inflammation and oxidative stress. HPE administration may thus be an effective approach to the treatment of cachexia, cardiac hypertrophy and fibrosis.

## 1 Introduction

Cachexia is a complex metabolic syndrome characterized by severe weight loss, undernutrition and exhaustion that accompanies chronic diseases such as cancer, heart failure and rheumatoid arthritis [1]. Although there is no clear definition of cachexia, reduction of lean body mass and elevation of protein degradation are diagnostic indicators. Abnormal acceleration of metabolism, which is represented by elevated resting energy expenditure, worsens the undernutrition. Unlike the weight loss caused by starvation, cachexia cannot be ameliorated through high calorie infusion. It has been suggested that chronic inflammation is the underlying cause of cachexia. At present, however, the precise mechanisms remain unknown, and cachexia remains an intractable metabolic disorder. Further study and identification of effective therapies are therefore essential.

Placenta embedding therapy began in the 1930s. Extraction of active ingredients from human placenta was established in the 1960s, and human placental extract (HPE) was later approved by the Food and Drug Administration for use in humans [2, 3, 4]. Multiple studies have shown that human placenta can serve as a source of numerous biologically active molecules [5, 6, 7]. Known biological effects of human placenta and HPE include, modulation of immune responses, protection and regeneration of hepatocytes, regulation of hormonal balance, effects on brain monoamine oxidase activity, anti-coagulation, facilitation of wound healing, and pigmentation [8, 9, 10, 11, 12, 13]. Studies using animal models have provided evidence that placenta extract improves liver function [14] and wound healing [15]. In clinical situations, HPE has been prescribed to treat chronic hepatitis, liver cirrhosis, viral hepatitis and other hepatic diseases. HPE is also used in the treatment of menopausal symptoms [10, 16].

Chronic infusion of angiotensin II (Ang II) into mice or rats causes body weight reduction that resembles cachexia and has been applied as a disease model of cachexia [17]. Cachexia often accompanies congestive heart failure (CHF) and it is called “cardiac cachexia”. The mechanisms of cardiac cachexia are poorly understood, but there is recent evidence that Ang II plays an important role; plasma Ang II level in patients with CHF associated with cachexia are higher than in patients without cachexia [18]. In the present study, therefore, we investigated the effects of HPE on the body weight and body composition of mice with Ang II-induced cachexia. Using this model, we also examined the effects of HPE on the cardiac hypertrophy, inflammation and fibrosis.

## 2 Material and method

### 2.1 Animals

Eight-week-old wild-type C57BL/6J male mice were purchased from a supplier of experimental animals (Charles river laboratories Japan, Inc. Kanagawa, Japan) and used for the study at 9-week-old. All mice were maintained according to a strict procedure under specific pathogen-free conditions in an environmentally controlled (12-h light/dark cycle; room temperature, 22 ± 2 °C) breeding room at the Division of Laboratory Animal Research, Department of Life Science, Research Center for Human and Environmental Sciences, Shinshu University. Before the surgical procedures, the mice were anesthetized through intraperitoneal injection of a combination of 0.3 mg/kg of medetomidine (Nippon Zenyaku Kogyo Co. Ltd., Koriyama, Japan), 4.0 mg/kg of midazolam (Astellas Pharma Inc. Tokyo, Japan) and 5.0 mg/kg of butorphanol (Meiji Seika Pharma Co. Ltd., Tokyo, Japan). All animal handling procedures were in accordance with a protocol approved by the Ethics Committee of Shinshu University School of Medicine.

### 2.2 Ang II infusion and HPE-treatment

Under anesthesia, we implanted an osmotic pump (Alzet model 1007D, DURECT Corporation, Cupertino, CA) under the dorsal skin of mice for subcutaneous infusion of Ang II (1 μg/kg/min; Sigma-Aldrich, MO) or control saline for 7 days. Mice were caged individually after pump implantation. Day 0 was defined as the start day of the Ang II infusion. The HPE used in this study was hydrolysate of human placenta (Laennec; Japan Bio Products Co., Ltd, Tokyo, Japan), which was administered by intramuscular injection of 3.6 mg/kg once a day for 7 days. Saline administered intramuscularly was used as the control. The first injection of HPE or control saline was administered immediately after osmotic pump implantation. Food intake and body mass were measured every day at around 10:00 am. To calculate adipose tissue weight, we combined the weights of the subcutaneous, epididymal, perirenal and mesenteric adipose tissue. Lean body mass weight was calculated by subtracting the adipose tissue weight from the total body weight.

### 2.3 Histology

Tissues were fixed overnight in 10% formalin, embedded in paraffin, and cut into 5-μm-thick sections for histological examination. The specimens were then deparaffinized for Masson trichrome (MT) staining. For immunohistochemistry, anti-αSMA, anti-CD45, anti-F4/80 and anti-CD3 antibodies were obtained from BD Biosciences (NJ). Nuclei were counterstained using DAPI (Life Technologies, CA). Fluorescence was observed using a fluorescence microscope equipped with the appropriate filter sets (BZ-900, KEYENCE, Osaka, Japan). Areas of interest were quantified using a BZ-H3C module (KEYENCE).

### 2.4 RNA extraction and quantitative real-time RT-PCR

Total RNA was extracted from tissues using TRI Reagent (Molecular Research Center, Inc., OH), after which the RNA was treated with DNA Free (Thermo Fisher Scientific, MA) to remove contaminating DNA and reverse transcribed using a Primescript RT reagent Kit (TaKaRa, Shiga, Japan). Quantitative real-time RT-PCR was carried out using an Applied Biosystems 7300 real-time PCR System with SYBR green (Toyobo, Osaka, Japan) or Realtime PCR Master Mix (Toyobo) and TaqMan probes (MBL, Nagano, Japan). The primers used are listed in Table 1. Values were normalized to mouse GAPDH (Pre-Developed TaqMan assay reagents, Thermo Fisher Scientific).

**Table 1:** Primers used for real-time PCR.

BNP	Forward	TCCAGAGCAATTCAAGATGCA
	Reverse	GTCTTTTCATTGCCGCTTCC
βMHC	Forward	GAGCTGTGGTGGCTTTTGTG
	Reverse	CGTCTGTCACTCAGTGCAGT
collagen α1	Forward	ATGGATTCCCGTTCGAGTACG
	Reverse	TCAGCTGGATAGCGACATCG
TGF-β	Forward	CCCGAAGCGGACTACTATGC
	Reverse	TAGATGGCGTTGTTGCGGT
MMP2	Forward	GTGACACCACGTGACAAGCC
	Reverse	TGGGAGCTCAGGCCAGAAT
ICAM-1	Forward	CCTAAAATGACCTGCAGACGG
	Reverse	TTTGACAGACTTCACCACCCC
PGC-1α	Forward	GGCACGCAGCCCTATTCA
	Reverse	CGACACGGAGAGTTAAAGGAAGA
PPARα	Forward	GGGATTGTGCACGTGCTTAA
	Reverse	TTTGGGAAGAGGAAGGTGTCA
MCAD	Forward	CACTTACTATGCCTCGATTGCAA
	Reverse	CGGCGTCAGTGGCTAGCT
ATP synthase	Forward	AGGCTATCTATGTGCCTGCTGAT
	Reverse	GCATCCAAATGGGCAAAGG
p67phox	Forward	CAGACCCAAAACCCCAGAAA
	Reverse	AAAGCCAAACAATACGCGGT
p47phox	Forward	ATCCTATCTGGAGCCCCTTGA
	Reverse	CACCTGCGTAGTTGGGATCC
p22phox	Forward	GGCCATTGCCAGTGTGATCT
	Reverse	GCTCAATGGGAGTCCACTGC
IL-6	Forward	CTGCAAGAGACTTCCATCCAGTT
	Reverse	GAAGTAGGGAAGGCCGTGG
IL-1β	Forward	CTACAGGCTCCGAGATGAACAAC
	Reverse	TCCATTGAGGTGGAGAGCTTTC
TNF-α	Forward	ACGGCATGGATCTCAAAGAC
	Reverse	AGATAGCAAATCGGCTGACG
MCP-1	Forward	GCAGTTAACGCCCCACTCA
	Reverse	CCTACTCATTGGGATCATCTTGCT
F4/80	Forward	GATGAATTCCCGTGTTGTTGGT
	Reverse	ACATCAGTGTTCCAGGAGACACA

### 2.5 Statistical analysis

Values are expressed as means ± SEM. One-way ANOVA was used to evaluate differences. Values of p < 0.05 were considered significant.

## 3 Results

### 3.1 Effect of HPE on body weight and body composition in mice treated with Ang II

**Fig. 1:**
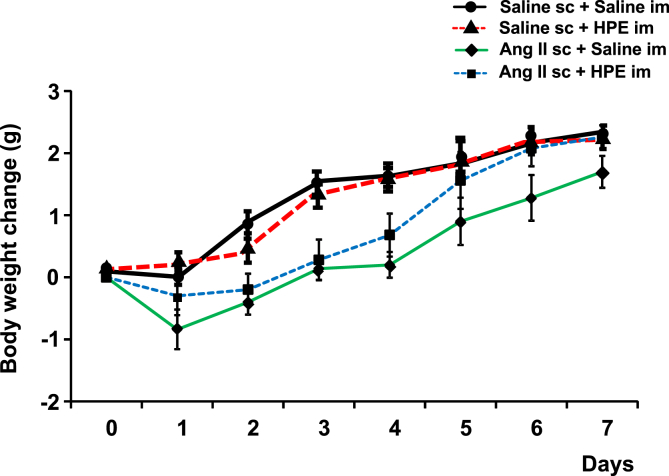
Effect of HPE on body weight in mice receiving Ang II infusion. Angiotensin II (Ang II) was subcutaneously (sc) infused (1 μg/kg/min) into mice for 7 days using osmotic pumps. Day 0 indicates the start day of Ang II infusion. Human placental extract (HPE) (Laennec; 3.6 mg/kg) or control saline was intramuscularly (im) administered daily for 7 days from day 0 to day 6 (Ang II sc + HPE im, Ang II sc + Saline im, respectively). Symbols are means ± SEM. n = 5.

**Fig. 2:**
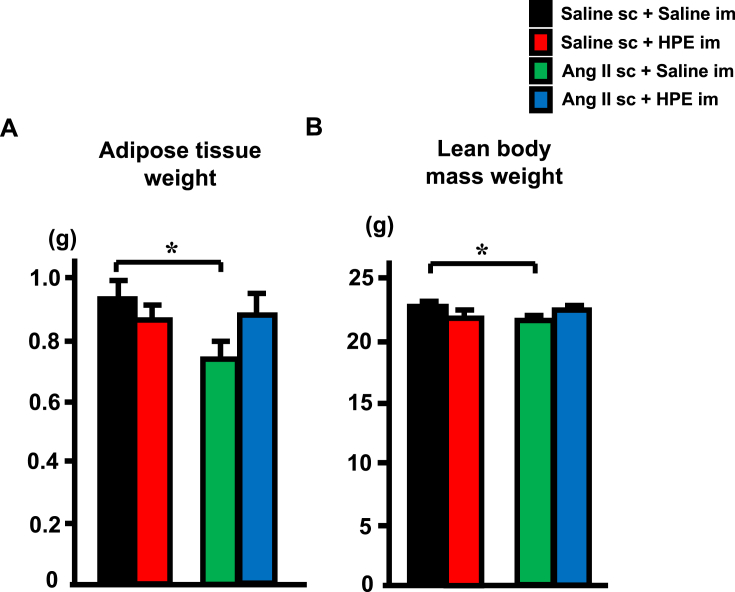
Effect of HPE on body weight composition in mice receiving Ang II infusion. A, Comparison of total (perirenal + mesenteric + epididymal + subcutaneous) white adipose tissue weights. B, Comparison of lean body mass weight (body weight - total adipose tissue weight). Mice were divided into subcutaneous infusion of control saline + intramuscular injection of control saline (Saline sc + Saline im), subcutaneous infusion of control saline + intramuscular injection of HPE (Saline sc + HPE im), subcutaneous infusion of Ang II + intramuscular injection of control saline (Ang II sc + Saline im) and subcutaneous infusion of Ang II + intramuscular injection of HPE (Ang II sc + HPE im) groups. Bars are means ± SEM. n = 5. *p < 0.05 between the indicated groups.

We first evaluated the effect of HPE on the body weights of mice in the Ang II-induced cachexia model (Fig. 1). Because we used 9-week-old male mice, which were still growing, body weights gradually increased throughout the observation period (day 0 to day 7). The continuous infusion of Ang II reduced that body weight gain, however. In particular, Ang II-infusion induced a transient reduction in body weight at day 1. HPE treatment suppressed the body weight reduction at day 1 and improved body weight gain thereafter. Ang II-infused mice also showed reduced food intake throughout the observation period, and this reduction was unaffected by HPE treatment (data not shown). Adipose tissue weight (Fig. 2A) and lean body mass weight (Fig. 2B) were both significantly lower in Ang II-treated mice than control mice on day 7. Although the difference was not statistically significant, Ang II with HPE showed higher tendency of adipose tissue weight and lean body mass weight compared to Ang II without HPE.

### 3.2 Effect of HPE on the heart weight

**Fig. 3:**
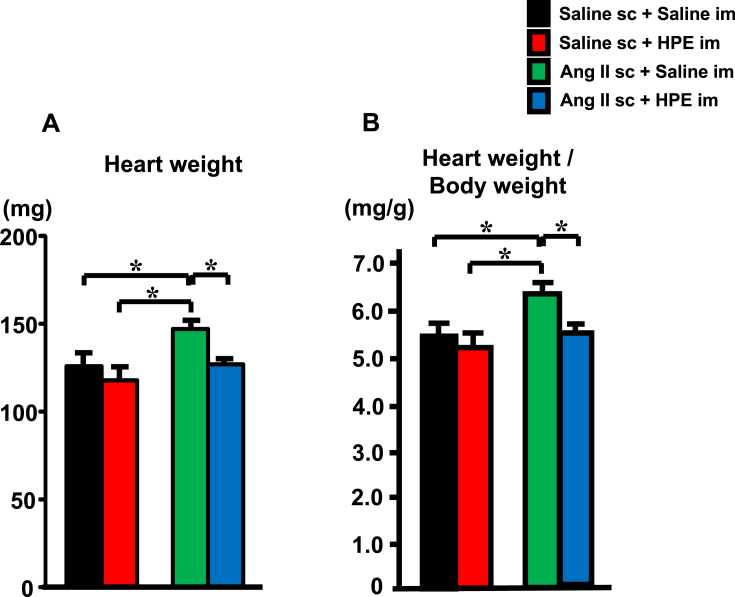
Effect of HPE-treatment on the heart weight. Heart weights (A) and heart weight/body weight ratios (B) on day 7 of Ang II infusion. Bars are means ± SEM. n = 5. *p < 0.05 between the indicated groups.

When liver and muscle weights were measured on day 7 of the Ang II infusion, we found that neither Ang II infusion nor HPE treatment significantly altered liver weight/body weight or soleus muscle weight/body weight ratios (data not shown). On the other hand, Ang II infusion, which causes cardiac hypertrophy, elevated both heart weights (Fig. 3A) and heart weight/body weight ratios (Fig. 3B). HPE treatment significantly suppressed the heart weight elevation caused by Ang II infusion.

### 3.3 Effects of HPE on cardiac remodeling-related gene expression

**Fig. 4:**
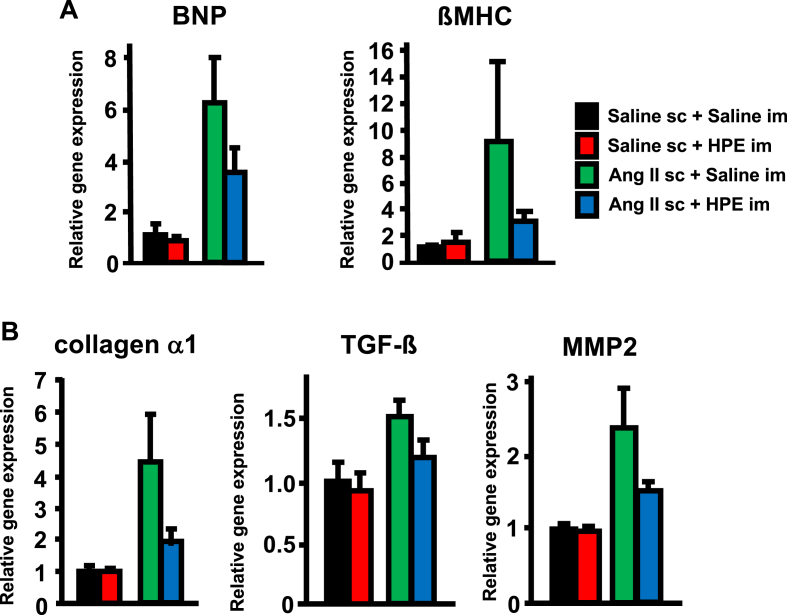
Effects of HPE on cardiac gene expression of heart failure and cardiac remodeling-related molecules. Relative gene expression of the indicated molecules related to heart failure (A) and cardiac remodeling (B) on day 7 of Ang II infusion. The expression level in the Saline sc + Saline im group was assigned a value of 1. Bars are means ± SEM. n = 5. *p < 0.05, **p < 0.01 between the indicated groups.

Based on the observation that HPE treatment may prevent the cardiac hypertrophy induced by continuous Ang II infusion, we analyzed cardiac gene expression on day 7. We initially assessed expression of heart failure-related molecules (Fig. 4A). Elevated BNP expression is diagnostic for heart failure, and myosin heavy chain isoform switch to βMHC is also observed in the failing heart. Although the difference was not statistically significant, Ang II infusion led to increases in cardiac expression of both BNP and βMHC genes, and this effect was suppressed by HPE treatment. We also assessed expression of molecules related to cardiac remodeling (Fig. 4B). Although the difference was not statistically significant, Ang II infusion upregulated expression of collagen α1, TGF-β and MMP2, all which are involved in mediating cardiac remodeling, and again that effect was suppressed by HPE.

### 3.4 Effects of HPE on cardiac hypertrophy and fibrosis

**Fig. 5:**
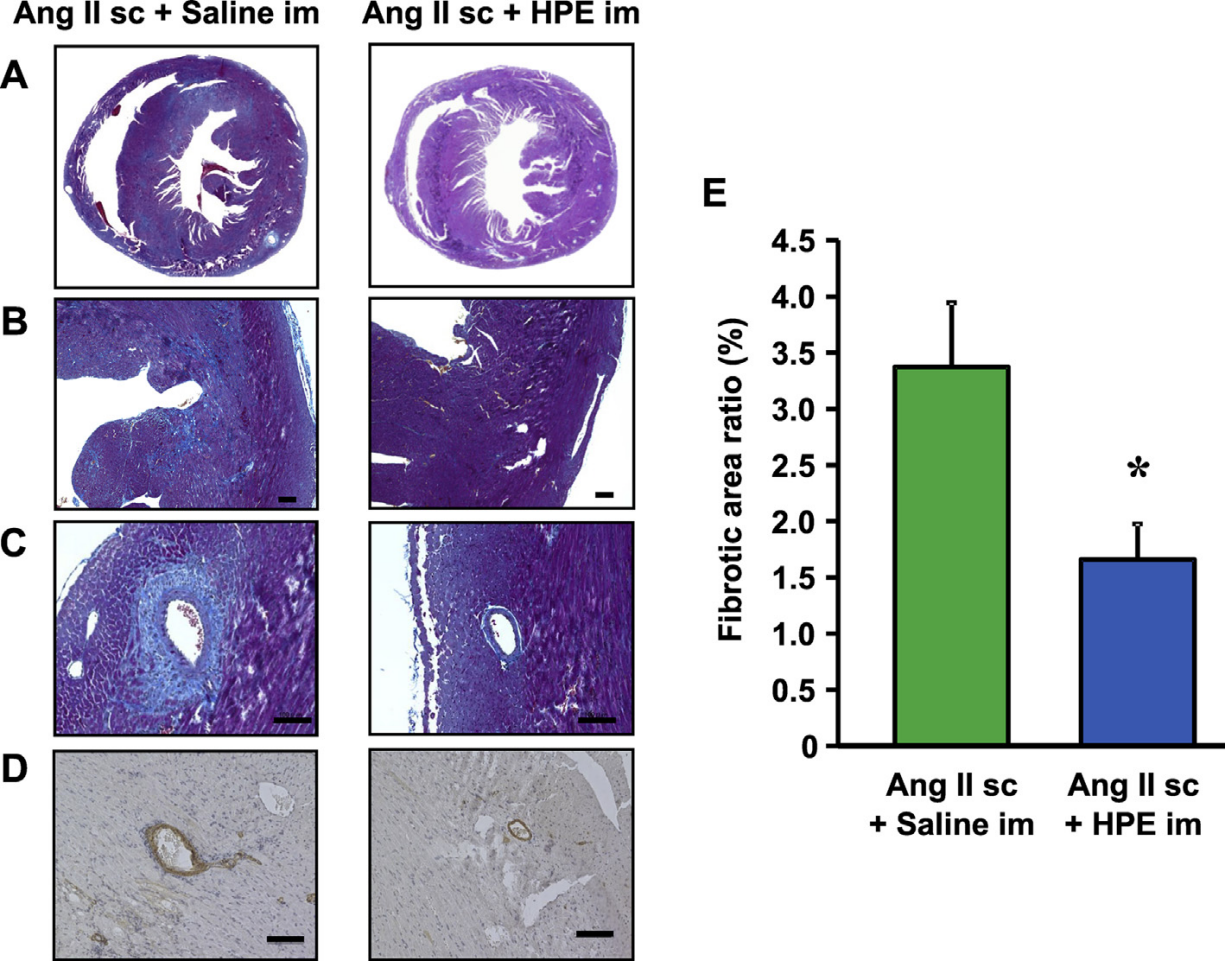
Effects of HPE on cardiac hypertrophy and fibrosis. A, Masson trichrome staining of short axial sections of whole hearts from mice infused for 7 days with Ang II, with and without HPE. B, Magnification of a part of the left ventricular wall. Blue staining indicates interstitial fibrosis. C, Masson trichrome staining showing perivascular fibrosis (blue staining) around the coronary artery. D, αSMA immunostaining in the left ventricular wall. Scale bars in B-D are 100 μm. **E,** Percent fibrotic area ratios. Fibrotic areas per microscope field were calculated at a magnification of 100x. Randomly selected fields from 5 sections from whole hearts per mouse were used for the analysis. Bars are means ± SEM. n = 4. *p < 0.05.

To further examine the cardiac effects of HPE, we performed a pathological analysis of the heart on day 7 of the Ang II infusion, with and without HPE. We found that HPE treatment prevented the cardiac hypertrophy caused by Ang II infusion (Fig. 5A). Masson trichrome staining of the left ventricular wall showed that Ang II infusion led to interstitial fibrosis (Fig. 5B) and perivascular fibrosis around the coronary artery (Fig. 5C). Expression of α-smooth muscle actin (αSMA) was detected in the activated cardiac myofibroblasts, which play critical roles in mediating cardiac fibrosis by producing extra cellular matrix [19]. Myofibroblasts positive for αSMA were detected around the coronary arteries of the Ang II-infused mice (Fig. 5D). In addition, HPE reduced the percent fibrotic area in sections of myocardial tissue (Fig. 5E).

### 3.5 Effects of HPE on cardiac expression of genes related to mitochondria, oxidative stress and inflammation

**Fig. 6:**
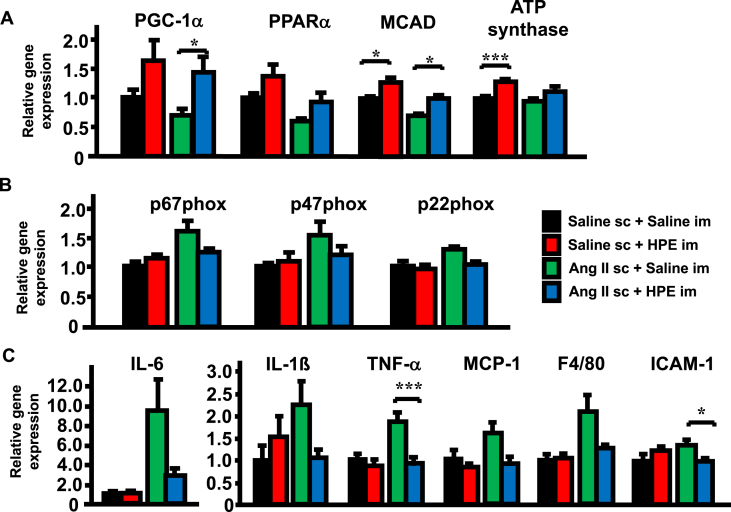
Effects of HPE on cardiac gene expression of mitochondria-related molecules, NADPH oxidase subunits and inflammation-related molecules. Relative gene expression of mitochondria-related molecules (A), NADPH oxidase subunits (B) and inflammation-related molecules (C) on day 7 of Ang II infusion are shown. The expression level in the Saline sc + Saline im group was assigned a value of 1. Bars are means ± SEM. n = 5. *p < 0.05, **p < 0.01, ***p < 0.001 between the indicated groups.

Mitochondria play central roles in energy metabolism in the heart, and mitochondrial dysfunction characterized by impaired bioenergetics and oxidative stress is a hallmark of heart failure [20]. We assessed the gene expression of mitochondria-related molecules on day 7 of the Ang II infusion. Compared to Ang II without HPE, Ang II with HPE increased expression of peroxisome proliferator-activated receptor γ coactivator 1-α (PGC-1α) and medium-chain acyl-CoA dehydrogenase (MCAD) (Fig. 6A). Interestingly, HPE upregulated MCAD and ATP synthase even in the absence of Ang II.

Because Ang II infusion is known to increase oxidative stress within the heart [21], we also analyzed the gene expression of NADPH oxidase, which is the major source of oxidative stress in the heart (Fig. 6B). Although the difference was not statistically significant, Ang II infusion enhanced expression of the NADPH oxidase subunits p67phox, p47phox and p22phox, and HPE treatment suppressed those effects. Ang II infusion is also known to induce inflammation within the heart [22]. Consistent with that, Ang II infusion showed tendency of the upregulation of the inflammation-related molecules IL-6, IL-1β, TNF-α, MCP-1, F4/80, and ICAM-1 (Fig. 6C). Among them, HPE treatment significantly suppressed the expression of TNF-α and ICAM-1.

### 3.6 Effects of HPE on inflammatory cell infiltration in the heart

**Fig. 7:**
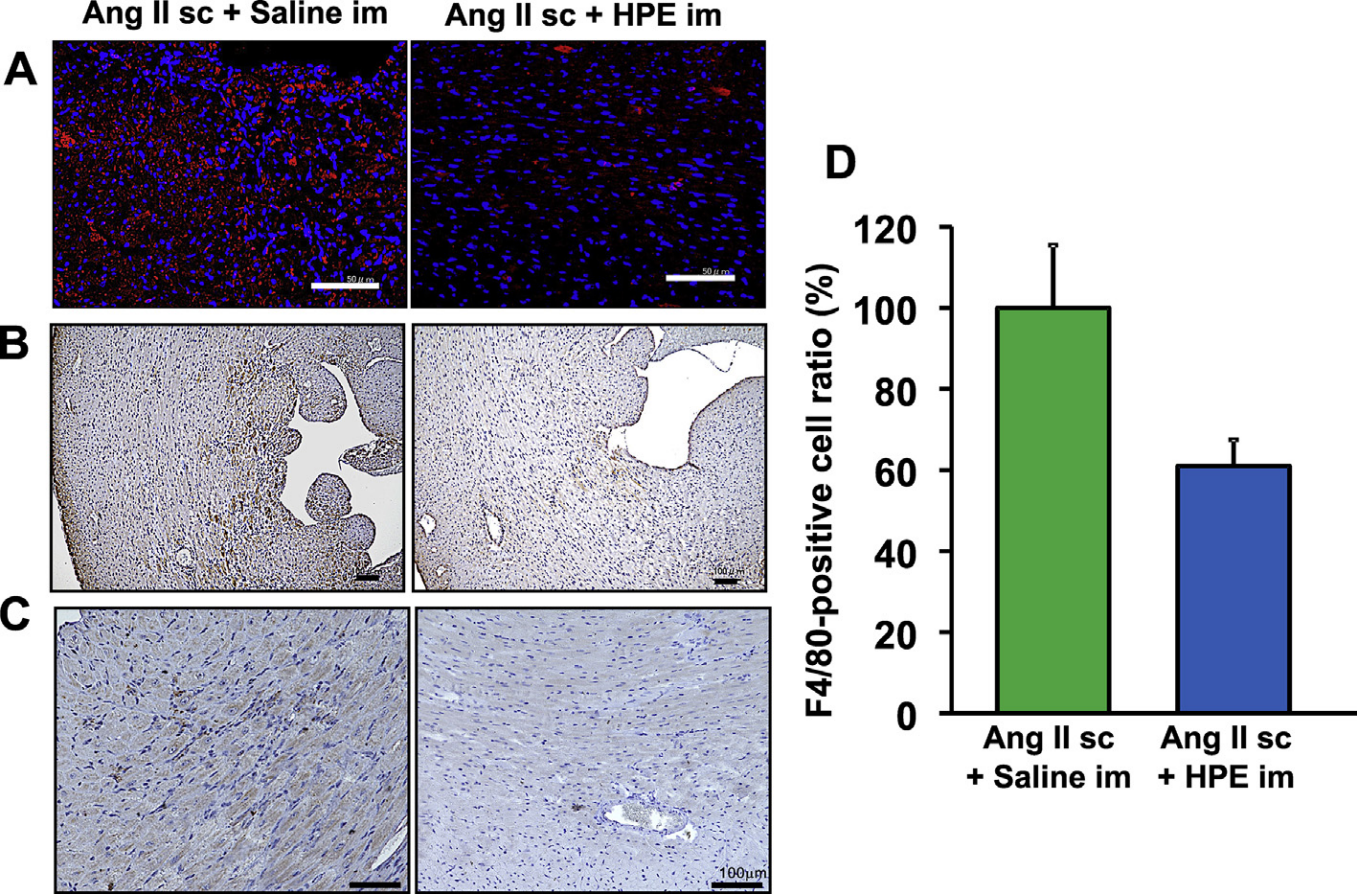
Effects of HPE on inflammatory cell infiltration of the heart immunostaining for inflammatory cell markers in sections of left ventricle from mice infused for 7 days with Ang II, with and without HPE. A, CD45 (pan leukocyte marker). B, F4/80 (macrophage marker). C, CD3 (T cell marker). Scale bars are 100 μm. D, Relative F4/80-positive cell numbers. Numbers of F4/80-positive cells in whole heart sections were counted. The cell number in the AngII sc + Saline im group was assigned a value of 100%. Bars are means ± SEM. n = 4.

Given that Ang II infusion upregulated expression of several inflammation-related molecules, and HPE prevented it, we analyzed the effects of HPE on Ang II-induced inflammatory cell infiltration of the heart. Seven days of Ang II infusion led to infiltration of inflammatory cells into the interstitial tissue of the left ventricular wall. Immunostaining revealed that these inflammatory cells were positive for CD45 (pan leukocyte marker (Fig. 7A)), F4/80 (macrophage marker (Fig. 7B)) or CD3 (T cell marker (Fig. 7C)). Although the difference was not statistically significant, HPE treatment showed tendency of the reduction of infiltrating inflammatory cells (Fig. 7D).

## 4 Discussion

Cachexia is an intractable metabolic disorder that causes extreme weight loss and muscle wasting. It is a symptom of many chronic diseases, including cancer, liver failure, congestive heart failure (CHF) and chronic kidney disease (CKD) [23]. Unlike the weight loss caused by starvation, cachexia cannot be ameliorated by high-calorie infusion and, up to now, no effective treatments other than anabolic steroids have been reported [24]. The mechanisms underlying cachexia are complex, but it is often accompanied by elevated angiotensin II (Ang II). Cachexic patients with advanced CHF and CKD often show increased Ang II levels, and administration of an angiotensin-converting enzyme inhibitor can mitigate the weight loss [25]. Moreover, it was found that Ang II infusion in rodents leads to skeletal muscle wasting [17].

HPE functions as a source of numerous biologically active molecules [5, 6, 7]. In clinical situations, HPE has been prescribed to treat chronic hepatitis, liver cirrhosis and other hepatic diseases, as HPE treatment improves liver function [26]. In an earlier study, we incidentally observed that HPE treatment led to body weight gain in a mouse model of chronic hepatitis. That observation prompted us to investigate the effects of HPE on cachexia using an Ang II infusion-induced mouse cachexia model in the present study. We found that HPE treatment preserved both fat mass and lean body mass and prevented weight loss, which was also reported with IGF-1 [27] or ghrelin [17] treatment. Using the Ang II-induced cachexia model, IGF-1 [28] or ghrelin [17] was previously shown to suppress body weight reduction and increase food intake volume. By contrast, we found that HPE, had no effect on food intake, though body weight reduction was suppressed. This suggests that in this cachexia model, the beneficial effect of HPE on adipose tissue mass and muscle mass is mediated through preservation of anabolism rather than elevation of appetite.

Ang II administration reportedly leads to weight loss through increased protein breakdown and reduced protein synthesis in both adipose tissue and skeletal muscle [29]. It was also recently reported that HPE significantly increases the viability of C2C12 skeletal muscle cells and reduces H_2_O_2_-stimulated cell death. In addition, HPE reduced mitochondria fission-related gene expression (Drp1 and BNIP3) and increased mitochondrial biogenesis via the Sirt1/AMPK/PGC-1α pathway and autophagy regulation [30]. Taken together, these findings suggest HPE exerts protective effects against adipose tissue and skeletal muscle atrophy through reduction of oxidative cell death.

The cardiac effects of ghrelin [17] are strikingly different from those of HPE. Interestingly, Ang II administration causes skeletal muscle atrophy but hypertrophy in the heart. In the Ang II infusion model, ghrelin did not affect heart weight [17], whereas HPE significantly suppressed cardiac hypertrophy. This strongly suggests that, in this cachexia model, the beneficial effects of HPE on the heart are not mediated by ghrelin.

Ang II plays a key role in the pathogenesis of CHF. Not only does it elevate blood pressure, it acts directly to induce cardiac hypertrophy and fibrosis, so-called cardiac cachexia. In the present study, HPE treatment suppressed the elevation in cardiac expression of BNP and βMHC genes induced by Ang II infusion, which suggests amelioration of the heart failure. In addition, HPE treatment suppressed Ang II-induced cardiac expression of the fibrotic markers collagen α1 and TGF-β as well as the interstitial and perivascular fibrosis otherwise caused by the Ang II administration.

Mitochondria are central to energy metabolism in the heart, and their malfunction is closely related to heart failure. Ang II infusion reportedly downregulates several mitochondria-related molecules [29]. PGC-1α is known to be a master regulator of mitochondrial biogenesis. We found that HPE treatment increased cardiac expression of PGC-1α. Moreover, HPE reversed the downregulation of PGC-1α expression by Ang II. PPARα is a transcription factor involved in regulating mitochondrial metabolism, while MCAD is an enzyme involved in mitochondrial fatty acid β-oxidation. Like PGC-1α, levels of PPARα and MCAD expression were elevated by HPE treatment, and their downregulation by Ang II was reversed. HPE also had similar effects on expression of mitochondrial ATP synthase. These results suggest that HPE enhances mitochondrial energy metabolism, which may partly explain its protective effect against heart failure.

Another beneficial effect of HPE is attributable to its anti-oxidative stress and anti-inflammatory actions. Ang II induces oxidative stress and inflammation, both of which are closely involved in the pathogenesis of heart failure and cardiac remodeling [21, 22]. Ang II infusion elevated expression of the NADPH oxidase subunits p67phox, p47phox and p22phox. HPE treatment suppressed that effect, which suggests HPE reduces the oxidative stress induced by Ang II. Ang II infusion also elevated cardiac expression of inflammation-related molecules such as TNF-α and ICAM-1, and this effect too was inhibited by HPE treatment. Furthermore, HPE suppressed Ang II-induced infiltration of the left ventricular wall by macrophages and T cells, which suggests HPE suppresses chronic inflammation within the heart. The immunomodulatory effects of HPE have been demonstrated in multiple studies. Consistent with the crucial role played by the placenta in the generation and maintenance of fetal-maternal tolerance, HPE exerts immuno-inhibitory effects through several mechanisms [31]. From a therapeutic perspective, the efficacy of HPE in the treatment of inflammation has been clearly demonstrated [31, 32].

A large number of growth factors, their receptors, and other biological mediators have been identified in HPE [5, 33, 34, 35, 36, 37]. Among these, JBP485 (cyclo-trans-4-L-hydroxyprolyl-L-serine) is a recently isolated dipeptide that reportedly diminishes corneal epithelial cell damage in a dry eye model [38]. Moreover, JBP485 also reportedly exerts a hepatoprotective effect in an immune-mediated, concanavalin A (Con A)-induced liver injury model in mice. JBP485 inhibited Con A-induced liver damage by suppressing excessive inflammation, oxidative stress and hepatocellular apoptosis [39]. JBP485 could thus be one of the molecules whose actions underlie the beneficial effects of HPE on various chronic diseases. We do not yet have information on the direct effects of JBP485 in cachexia or heart failure, but it is an attractive target for future study.

Ang-II-induced cachexia is a well-known classical rodent model; however, a limitation of this study is that we analyzed changes occurring during a relatively short period of time. In future studies, we plan to analyze disease models that entail longer periods of observation to further clarify the beneficial effects of HPE in chronic diseases.

HPE has been used clinically for many years, and it appears safe without apparent major side effects. Our findings suggest HPE administration may be an effective approach to the treatment of cachexia. Further study about the comparison between HPE treatment and other previously reported remedies (IGF-1 and ghrelin) could help to define its therapeutic value for the treatment of cachexia.

## Declarations

### Author contribution statement

Akihiro Yamauchi: Conceived and designed the experiments; Performed the experiments; Analyzed and interpreted the data; Wrote the paper.

Akiko Kamiyoshi: Performed the experiments; Analyzed and interpreted the data.

Takayuki Sakurai: Performed the experiments.

Hiroyuki Miyazaki, Eiichi Hirano: Conceived and designed the experiments; Analyzed and interpreted the data; Contributed reagents, materials, analysis tools or data.

Hong Seok Lim, Taiichi Kaku: Contributed reagents, materials, analysis tools or data.

Takayuki Shindo: Conceived and designed the experiments; Analyzed and interpreted the data; Wrote the paper.

### Funding statement

This work was supported by Japan Bio Products Co., Ltd., as a collaborative project.

### Competing interest statement

The authors declare the following conflict of interests: Taiichi Kaku is a stockholder of Japan Bio Products Co., Ltd. Akihiro Yamauchi, Hiroyuki Miyazaki, Eiichi Hirano and Hong Seok Lim are employers of Japan Bio Products Co., Ltd.

### Additional information

No additional information is available for this paper.
